# Activation of AIM2 by hepatitis B virus results in antiviral immunity that suppresses hepatitis C virus during coinfection

**DOI:** 10.1128/jvi.01090-23

**Published:** 2023-10-03

**Authors:** Yongqi Li, Yang Yang, Tianyang Li, Zhengmin Wang, Chunfeng Gao, Rilin Deng, Faxiang Ma, Xinyang Li, Licong Ma, Renyun Tian, Huiyi Li, Haizhen Zhu, Lei Zeng, Yanhang Gao, Guoyue Lv, Junqi Niu, Ian Nicholas Crispe, Zhengkun Tu

**Affiliations:** 1 Institute of Translational Medicine, The First Hospital of Jilin University, Changchun, Jilin, China; 2 Hunan Provincial Key Laboratory of Medical Virology, State Key Laboratory of Chemo/Biosensing and Chemometrics, Institute of Pathogen Biology and Immunology of College of Biology, Hunan University, Changsha, Hunan, China; 3 Institute of Liver Diseases, The First Hospital of Jilin University, Changchun , Jilin, China; 4 Department of Laboratory Medicine and Pathology, University of Washington, Seattle, Washington, USA; University of Southern California, Los Angeles, California, USA

**Keywords:** hepatitis B virus, hepatitis C virus, NK cells, monocytes, AIM2

## Abstract

Hepatitis B virus (HBV) infection in immunocompetent adults is mostly self-limiting, whereas Hepatitis C virus (HCV) infection more often becomes chronic, suggesting that HBV is more susceptible to the host immune response. It is unclear how HBV induces effective antiviral immunity, but HBV also causes anti-HCV immunity leading to lower HCV viral loads in co-infected patients. Here, we found that a pathway in which HBV dsDNA is sensed by the inflammasome protein absent in melanoma-2, leading to caspase-1 activation in monocytes, cleavage of pro-IL-18 and secretion of IL-18 protein, activation of NK cells, and their synthesis of interferon-γ. Such activation could suppress not only HBV but also HCV replication, suggesting a mechanism through which the two viruses could engage in cross-talk during coinfection.

**IMPORTANCE:**

Clinical data suggest that Hepatitis C virus (HCV) levels are generally lower in Hepatitis B virus (HBV) co-infected patients, but the mechanism is unknown. Here, we show that HBV, but not HCV, activated absent in melanoma-2. This in turn results in inflammasome-mediated cleavage of pro-IL-18, leading to an innate immune activation cascade that results in increased interferon-γ, suppressing both viruses.

## INTRODUCTION

Hepatitis B virus (HBV) is a DNA virus that infects over 360 million people worldwide, despite an available vaccine. Current treatment options for HBV infection are limited and rarely result in clearance (1). Hepatitis C virus (HCV) is an RNA virus that infects 71 million people worldwide, with no available vaccine but successful treatment options through direct acting antiviral (DAA) agents (2). Replicating in hepatocytes and targeting the liver, both HBV and HCV can cause hepatitis that may progress to cirrhosis or hepatocellular carcinoma (3). HCV infection induces innate immune activation in hepatocytes (4), but multiple viral mechanisms impede the development of effective antiviral immunity (5, 6), resulting in persistent infection (3). In contrast, HBV infection does not cause strong innate immune activation in hepatocytes (7, 8), but most immunocompetent individuals can still eliminate the virus (3). This work provides an explanation for this paradox.

HBV and HCV frequently coexist in highly endemic areas due to shared modes of transmission, despite different replication strategies and life cycles (9). The two viruses exhibit immunological crosstalk (10). Thus, the suppression of HBV replication occurred in chronic hepatitis B patients who developed acute hepatitis C (11). Conversely, clearing HCV with DAA treatment reduced hepatic interferon (IFN) response, leading to HBV reactivation in coinfected individuals (12). Similarly, HBV superinfection can inhibit HCV replication (13) and may even result in spontaneous clearance of HCV (14). In a study of HBV/HCV coinfected subjects, we found lower HCV viral loads compared to HCV-only infected individuals (15). Studies have shown that HBV and HCV can replicate in the same hepatocyte without evidence for direct interference *in vitro* (16 – 19). Therefore, HBV-induced anti-HCV immunity is probably mediated through immune cells. In this study, we found evidence that HBV-induced antiviral effects were dependent on NK cell IFN-γ production, which was activated by IL-18 from monocytes. However, how HBV induces IL-18 production of monocytes to result in antiviral effect of NK cells has remained obscure.

The absent in melanoma 2 (AIM2) protein is a cytosolic double-stranded DNA (dsDNA) receptor that contributes to the host defense against bacterial and viral pathogens. This sensor binds to DNA via its HIN200 domain and oligomerizes with ASC to initiate the formation of a caspase-1-activating inflammasome, leading to the secretion of proinflammatory cytokines, including IL-1β and IL-18 (20, 21). Like many inflammasome elements, AIM2 is strongly expressed in monocytes. In HBV infection, the expression of AIM2 in peripheral blood monocytes differs in acute versus chronic hepatitis, while in the context of chronic HBV, higher levels of AIM2 correlate with increased inflammation (22 – 24), but the mechanism is unclear. Since HBV infection involves both incomplete dsDNA viral genomes and quiescent covalently closed circular DNA (cccDNA), it is realistic to propose that HBV DNA sensing via AIM2 will lead to inflammasome activation, with the potential to activate downstream antiviral immunity.

In this study, we demonstrate that both HBV and HCV activated NF-κB leading to the expression of pro-IL-18 in monocytes, but only HBV dsDNA was recognized by AIM2, with the activated inflammasome leading to caspase-1 activity, pro-IL-18 cleavage, and IL-18 secretion, which in turn induced NK cell IFN-γ production that mediated the antiviral effect.

## RESULTS

### HBV induces an antiviral effect of peripheral blood mononuclear cells, but HCV does not

**Fig 1:**
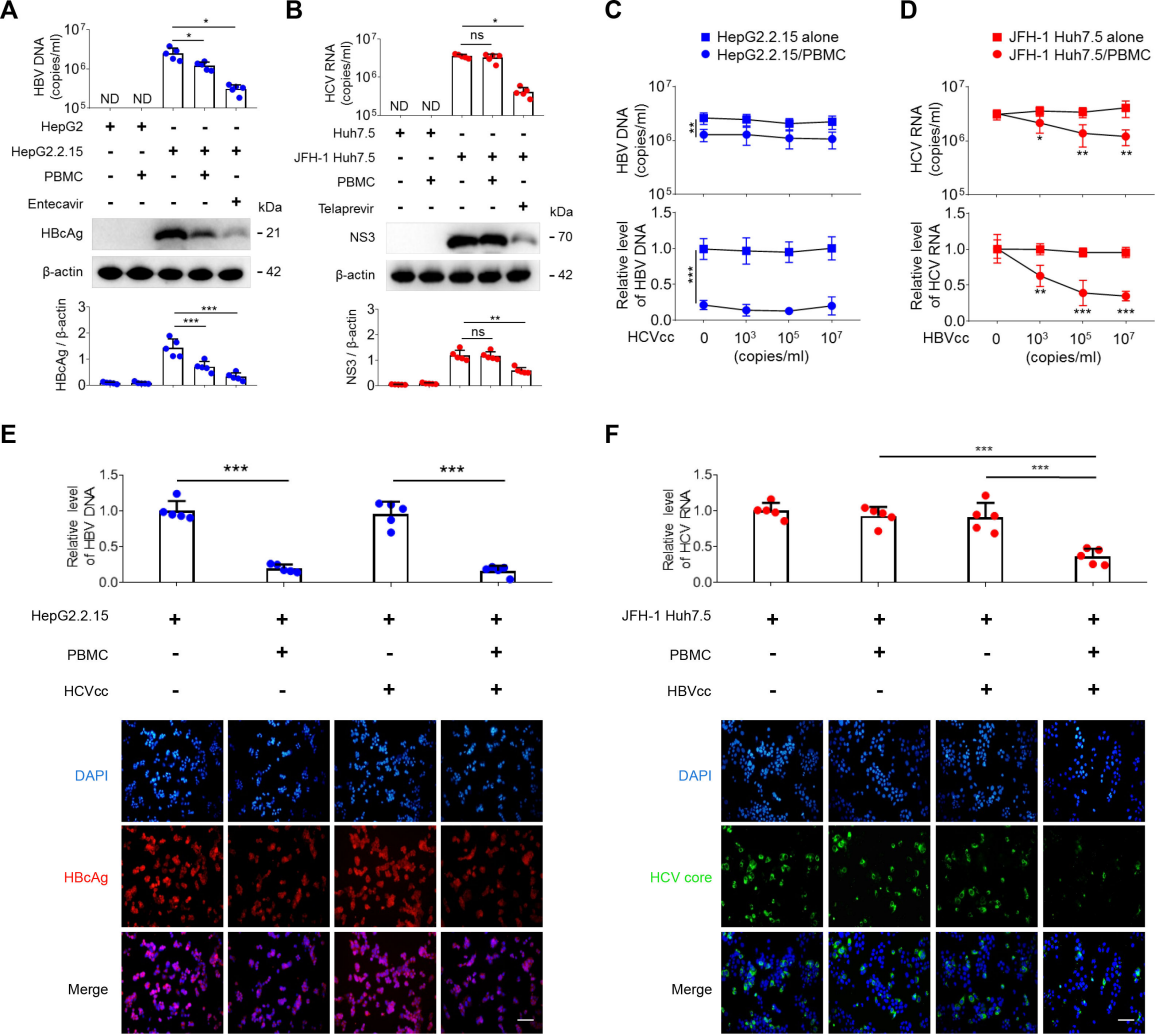
HBV induces an antiviral effect in peripheral blood mononuclear cells, but HCV does not. HepG2.2.15 cells or JFH-1 Huh7.5 cells were seeded and cultured with or without entecavir (10 µM) or telaprevir (5 µM) for 48 h and then co-cultured in trans-well with or without human PBMCs from healthy donors at an E:T ratio of 20:1 for 48 h. The levels of extracellular HBV-DNA and HCV-RNA were examined by qPCR and qRT-PCR (A and B, upper panel), and the expressions of HBcAg and HCV-NS3 were examined by Western blot (A and B, lower panel). HepG2.2.15 cells or JFH-1 Huh7.5 cells were co-cultured in trans-wells with or without PBMCs in the presence of increasing concentrations of HCVcc (0, 10^3^, 10^5^, and 10^7^ copies/mL) or HBVcc (0, 10^3^, 10^5^, and 10^7^ copies/mL) for 48 h, qPCR for extracellular and intracellular HBV-DNA (**C**), qRT-PCR for extracellular and intracellular HCV-RNA (**D**). HepG2.2.15 cells or JFH-1 Huh7.5 cells were co-cultured in trans-wells with or without PBMCs in the presence of HCVcc (10^5^ copies/mL) or HBVcc (10^5^ copies/mL) for 48 h; intracellular HBV DNA and HBcAg (**E**) and intracellular HCV RNA and HCV core (**F**) expressions were detected by qPCR or qRT-PCR and immunofluorescence. Scale bar = 100 µm. Mean with SD and *P*-value are displayed. *n* = 5 per group. Statistical significance was calculated using one-way ANOVA. *P < 0.05, **P < 0.01, ***P < 0.001. *ND*, not detectable. *ns*, not significant.

We first compared the HBV- and HCV-induced antiviral effect in peripheral blood mononuclear cells (PBMCs). Such cells from the blood of healthy donors were co-cultured in trans-wells with HBV- and HCV-secreting cell lines (HepG2.2.15 and JFH-1 Huh7.5), HepG2 and Huh7.5 as negative controls, HepG2.2.15 treatment with entecavir and JFH-1 Huh7.5 treatment with telaprevir as antiviral positive controls, respectively. The trans-well co-culture models excluded the possibility that the observed effects of peripheral blood immune cells on HepG2.2.15 and JFH-1 Huh7.5 are due to histo-incompatibility. Both intracellular and extracellular viral markers were analyzed. We observed that while entecavir and telaprevir effectively inhibited the viral replication in HepG2.2.15 and JFH-1 Huh7.5, respectively, the expression of HBcAg and the level of HBV-DNA were inhibited in HepG2.2.15 cells co-cultured with PBMCs (Fig. 1A), but the expression of HCV-NS3 and the level of HCV-RNA were not affected in JFH-1 Huh7.5 cells co-cultured with PBMCs (Fig. 1B).

To further test whether HBV and HCV reciprocally influenced the host immune response and viral replication, PBMCs were co-cultured in trans-wells with HepG2.2.15 cells or JFH-1 Huh7.5 cells in the presence of HCVcc (cell culture-derived HCV) or HBVcc (cell culture-derived HBV). We observed that although HBVcc couldn’t infect and replicate in the PBMCs (Fig. S1), HBVcc caused a dose-dependent inhibition of HCV replication in JFH-1 Huh7.5 cells co-cultured with PBMCs (Fig. 1D), while HCVcc did not affect HBV replication in HepG2.2.15 cells co-cultured with PBMCs (Fig. 1C); 10^5^ copies/mL of HCVcc and HBVcc were the optimal concentrations since higher concentrations of HBVcc did not further increase the inhibition and were used as the standard concentrations in subsequent experiments. Using immunofluorescence and flow cytometry, HBcAg expression was suppressed when HepG2.2.15 cells were co-cultured in trans-wells with PBMCs in both the presence and absence of HCVcc (Fig. 1E; Fig. S2A), while HCV core expression was suppressed when JFH-1 Huh7.5 cells were similarly co-cultured with PBMCs in the presence of HBVcc (Fig. 1F; Fig. S2B).

Taken together, these results indicate that HBV virions directly induced an antiviral effect that suppressed HBV replication, but HCV did not induce a similar effect.

### HBV-induced, but not HCV-induced, antiviral effects were due to IFN-γ, which is produced by NK cells

**Fig 2:**
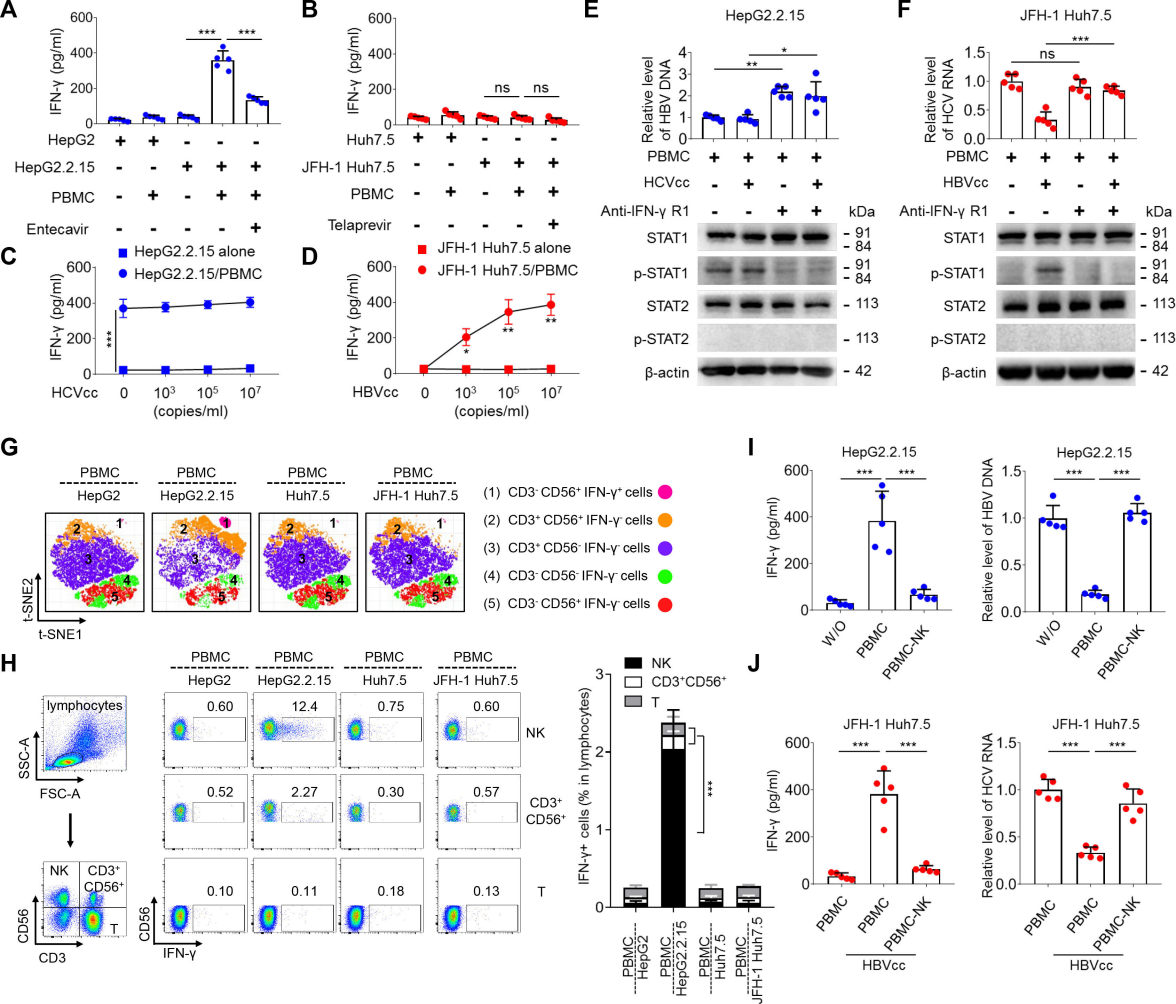
HBV-induced but not HCV-induced NK cells produce IFN-γ for antiviral effect. HepG2.2.15 cells or JFH-1 Huh7.5 cells were seeded and cultured with or without entecavir (10 µM) or telaprevir (5 µM) for 48 h and then co-cultured in trans-well with or without human PBMCs from healthy donors at an E:T ratio of 20:1 for 48 h. IFN-γ production was examined by ELISA in supernatants (**A and B**). HepG2.2.15 cells or JFH-1 Huh7.5 cells were co-cultured in trans-wells with or without PBMCs in the presence of increasing concentrations of HCVcc (0, 10^3^, 10^5^, and 10^7^ copies/mL) or HBVcc (0, 10^3^, 10^5^, and 10^7^ copies/mL) for 48 h; IFN-γ production was examined by ELISA in supernatants (**C and D**). HepG2.2.15 cells or JFH-1 Huh7.5 cells were co-cultured in trans-wells with PBMCs in the presence or absence of HCVcc (10^5^ copies/mL), HBVcc (10^5^ copies/mL), and anti-IFN-γR1 Ab (2 µg/mL). The intracellular HBV-DNA or HCV-RNA was assayed for 48 h by qPCR or qRT-PCR (E and F, upper panel), and the expression of IFN signal proteins was examined for 3 h by Western blot (E and F, lower panel). HepG2.2.15 cells (HepG2) or JFH-1 Huh7.5 cells (Huh7.5) were co-cultured in trans-wells with human PBMCs for 24 h, and the expression of intracellular IFN-γ in PBMCs cell subsets was analyzed by t-SNE (**G**) and flow cytometry (**H**). HepG2.2.15 cells or JFH-1 Huh7.5 cells were co-cultured in trans-wells with PBMCs and NK-depleted PBMCs in the presence of HBVcc for 48 h, IFN-γ production was detected by ELISA (I and J, left panel), and intracellular HBV-DNA or HCV RNA was assayed by qPCR or qRT-PCR (I and J, right panel). Mean with SD and *P*-value are displayed. *n* = 5 per group. Statistical significance was calculated using one-way ANOVA. *P < 0.05, **P < 0.01, ***P < 0.001. *ns*, not significant.

IFN-γ plays an important role in immunity against viral replication (25, 26). We first tested if PBMCs co-cultured in trans-wells with HepG2.2.15 cells or JFH-1 Huh7.5 cells produced IFN-γ. The level of IFN-γ in the supernatants was measured using ELISA. As expected, PBMCs co-cultured with HepG2.2.15 cells produced significantly higher IFN-γ than PBMCs co-cultured with HepG2 cells, and entecavir obviously inhibited IFN-γ production in PBMCs co-cultured with HepG2.2.15 cells (Fig. 2A). This result suggests that HBV virions released by HepG2.2.15 cells are responsible for triggering an immune response. Neither PBMCs co-cultured with JFH-1 Huh7.5 cells nor PBMCs co-cultured with Huh7.5 cells secreted IFN-γ (Fig. 2B). The level of IFN-γ was not detectable in cell cultures of HepG2.2.15 and JFH-1 Huh7.5. HBVcc caused a dose-dependent increase of IFN-γ production in PBMCs co-cultured with JFH-1 Huh7.5 (Fig. 2D), while HCVcc did not affect IFN-γ production in PBMCs co-cultured with HepG2.2.15 (Fig. 2C).

Different classes of IFN exert different biological activities through different signal transduction pathways. IFN-γ mainly promotes STAT1 phosphorylation and forms homodimers, whereas IFN-α leads to STAT1-STAT2 heterodimer formation (27). To determine whether HBV-induced IFN-γ production contributes to the antiviral effect, we added anti-IFN-γR1 Ab to the co-cultures of PBMCs with HepG2.2.15 cells or JFH-1 Huh7.5 cells in the presence or absence of HBVcc or HCVcc. Intracellular HBV-DNA or HCV-RNA was examined by qPCR or qRT-PCR, and p-STAT1(Y701) and p-STAT2(Y690) were detected by Western blot in HepG2.2.15 and JFH-1 Huh7.5 cells. We observed that the blockade of IFN-γ signaling increased the HBV replication and decreased the phosphorylation level of STAT1 Y701 in PBMCs co-cultures with HepG2.2.15 in the presence or absence of HCVcc (Fig. 2E). When JFH-1 Huh7.5 cells were co-cultured in trans-wells with PBMCs in the presence of HBVcc, HCV replication was suppressed and phosphorylation of STAT1 was activated, and the blockade of IFN-γ signaling restored HCV replication, but decreased the phosphorylation of STAT1 (Fig. 2F). Among all the cell co-cultures, phosphorylation of STAT2 was not detectable.

To identify which cell subset(s) in peripheral blood contributes to IFN-γ production, human PBMCs were co-cultured in trans-wells with HepG2.2.15 cells or JFH-1 Huh7.5 cells. The cell surface and intracellular IFN-γ staining of PBMCs were examined by flow cytometry. We applied a dimensional reduction analysis to the FACS data, which identified one cluster of IFN-γ-produced cells based on the expression of CD3, CD56, cellular size, and granularity. Of note, CD3^-^CD56^+^IFN-γ^+^ NK cells were exclusively found in PBMCs co-cultures with HepG2.2.15 cells (Fig. 2G). We reached the same conclusion using representative dot plots and statistical analysis using a gating strategy (Fig. 2H). To further test if NK cells were the main cell subset to produce IFN-γ mediating the antiviral effect, PBMCs and NK-depleted PBMCs were co-cultured in trans-wells with HepG2.2.15 or JFH-1 Huh7.5 cells in the presence of HBVcc. IFN-γ in the supernatants was detected using ELISA, and intracellular HBV DNA and HCV RNA levels of HepG2.2.15 and JFH-1 Huh7.5 cells were examined by qPCR and qRT-PCR. We observed that the depletion of NK cells inhibited IFN-γ production and restored HBV and HCV replication when PBMCs were co-cultured with HepG2.2.15 (Fig. 2I) and with JFH-1 Huh7.5 cells in the presence of HBVcc (Fig. 2J), respectively. Thus, we concluded that HBV-induced but not HCV-induced NK cells produce IFN-γ which mediates the antiviral effect.

### HBV-induced NK cells produce IFN-γ via IL-18 secreted by monocytes

**Fig 3:**
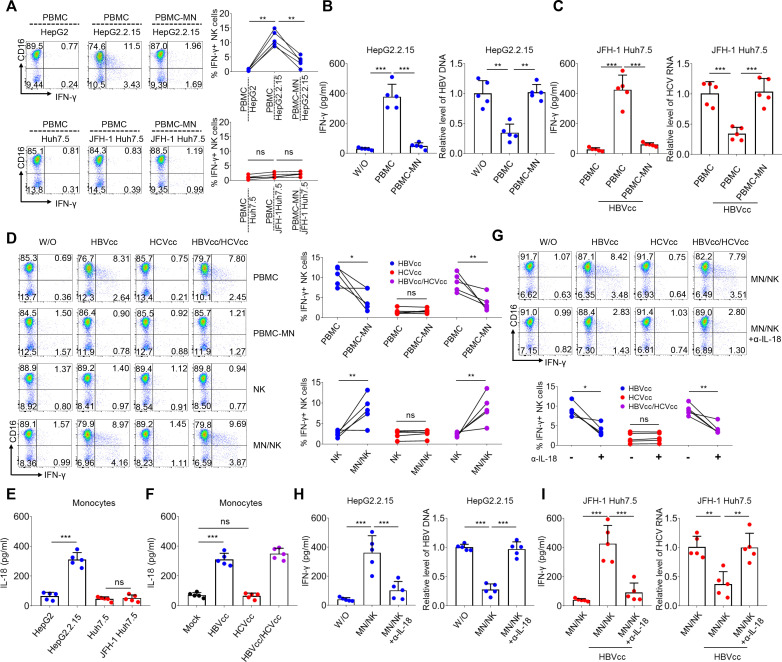
HBV-induced monocytes produce IL-18 to activate NK cells. HepG2.2.15 or JFH-1 Huh7.5 cells were co-cultured in trans-wells with PBMCs and PBMCs depleted of monocytes for 24 h, NK cell IFN-γ production was examined by intracellular cytokine staining (**A**). PBMCs and PBMCs depleted of monocytes were co-cultured in trans-wells with HepG2.2.15 or JFH-1 Huh7.5 cells in the presence of HBVcc for 48 h, IFN-γ was detected by ELISA (B and C, left panel), and intracellular HBV-DNA or HCV-RNA was assayed by qPCR or qRT-PCR (B and C, right panel). Freshly isolated human PBMCs, PBMCs depleted of monocytes, purified NK cells, and purified monocytes were cultured or co-cultured in the presence of HBVcc (10^5^ copies/mL), HCVcc (10^5^ copies/mL), or both HBVcc and HCVcc together for 24 h. The number ratio of monocytes to NK cells is 1:1, and intracellular IFN-γ production in NK cells was measured by flow cytometry (**D**). Purified monocytes from healthy donors were trans-wells co-cultured with HepG2.2.15 (HepG2) cells or JFH-1 Huh7.5 (Huh7.5) cells, or stimulated with HBVcc and/or HCVcc; IL-18 expression in the supernatant was detected for 24 h by ELISA (**E and F**). NK cells and monocytes from healthy controls were co-cultured with HBVcc and/or HCVcc in the presence of 10 µg/mL anti-IL-18 for 24 h. The number ratio of monocytes to NK cells is 1:1, and NK cell IFN-γ production was examined by intracellular cytokine staining (**G**). HepG2.2.15 or JFH-1 Huh7.5 cells were co-cultured in trans-wells with monocytes and NK cells in the presence of anti-IL-18 for 48 h, IFN-γ production was detected by ELISA (H and I, left panel), and intracellular HBV-DNA or HCV-RNA was assayed by qPCR or qRT-PCR (H and I, right panel). Mean with SD and *P*-value are displayed. *n* = 5 per group. Statistical significance was calculated using one-way ANOVA, except in D, E, and G, which were assessed by *t*-test. *P < 0.05, **P < 0.01, ***P < 0.001. *ns*, not significant.

Next, we investigated how HBV induces NK cells to produce IFN-γ. Total PBMCs and monocyte-depleted PBMCs were co-cultured in trans-wells with HepG2.2.15 or JFH-1 Huh7.5 cells in the presence or absence of HBVcc, the expression of CD3 and CD56 was used to gate on NK cells, and IFN-γ expression was determined by intracellular cytokine staining. IFN-γ secretion was detected using ELISA, and intracellular HBV DNA and HCV RNA levels of HepG2.2.15 cells and JFH-1 Huh7.5 cells were measured by qPCR and qRT-PCR. The results showed that IFN-γ expression in NK cells from monocytes-depleted PBMCs was significantly lower than that of NK cells from total PBMCs in co-culture with PBMCs and HepG2.2.15 (Fig. 3A). In addition, the depletion of monocytes from PBMCs restored HBV replication in PBMCs and HepG2.2.15 in co-culture (Fig. 3B), as well as HCV replication in PBMCs and JFH-1 Huh7.5 cells co-cultured with HBVcc (Fig. 3C).

To further test if HBV and/or HCV induced monocytes to interact with NK cells leading to IFN-γ production, freshly isolated human PBMCs, PBMCs depleted of monocytes, purified NK cells, and purified monocytes were cultured or co-cultured with HBVcc, HCVcc, or both HBVcc and HCVcc together. The effects of HBVcc and/or HCVcc on IFN-γ expression by NK cells are shown in Fig. 3D. On exposure to HBVcc and HBVcc/HCVcc, IFN-γ expression of NK cells from monocyte-depleted PBMCs was significantly lower than that of NK cells from total PBMCs. Pure NK cells failed to respond to both HBVcc and HCVcc, and NK cells co-cultured with monocytes secreted IFN-γ in response to HBVcc and HBVcc/HCVcc but not to HCVcc alone.

IL-18 is a key mediator of NK cell activation during NK cell interaction with monocytes and Kupffer cells as described previously (28). We first assessed IL-18 synthesis by monocytes in response to HBV and HCV using ELISA. Figure 3E and F depicts the IL-18 secretion by purified monocytes caused by HepG2.2.15 cells and HBVcc, but not JFH-1 Huh7.5 cells and HCVcc. To further determine whether HBV-induced IL-18 synthesis of monocytes activated NK cell IFN-γ production, anti-IL-18 antibody was added to the co-cultures of monocyte and NK cells with HBVcc and/or HCVcc. A representative experiment is shown in Fig. 3G. As expected, the blockade of IL-18 inhibited IFN-γ expression of NK cells in monocyte and NK cell co-cultured with HBVcc and HBVcc/HCVcc. The blockade of IL-18 restored HBV and HCV replication when monocyte and NK cell were co-cultured in trans-wells with HepG2.2.15 cells (Fig. 3H) and with JFH-1 Huh7.5 cells in the presence of HBVcc (Fig. 3I), respectively.

These results argue that HBV-induced NK cells produce IFN-γ in a monocyte-dependent manner, and IL-18 synthesis of monocytes by HBV is involved in NK cell IFN-γ production.

### HBV induces monocytes IL-18 production by activating the AIM2 inflammasome

**Fig 4:**
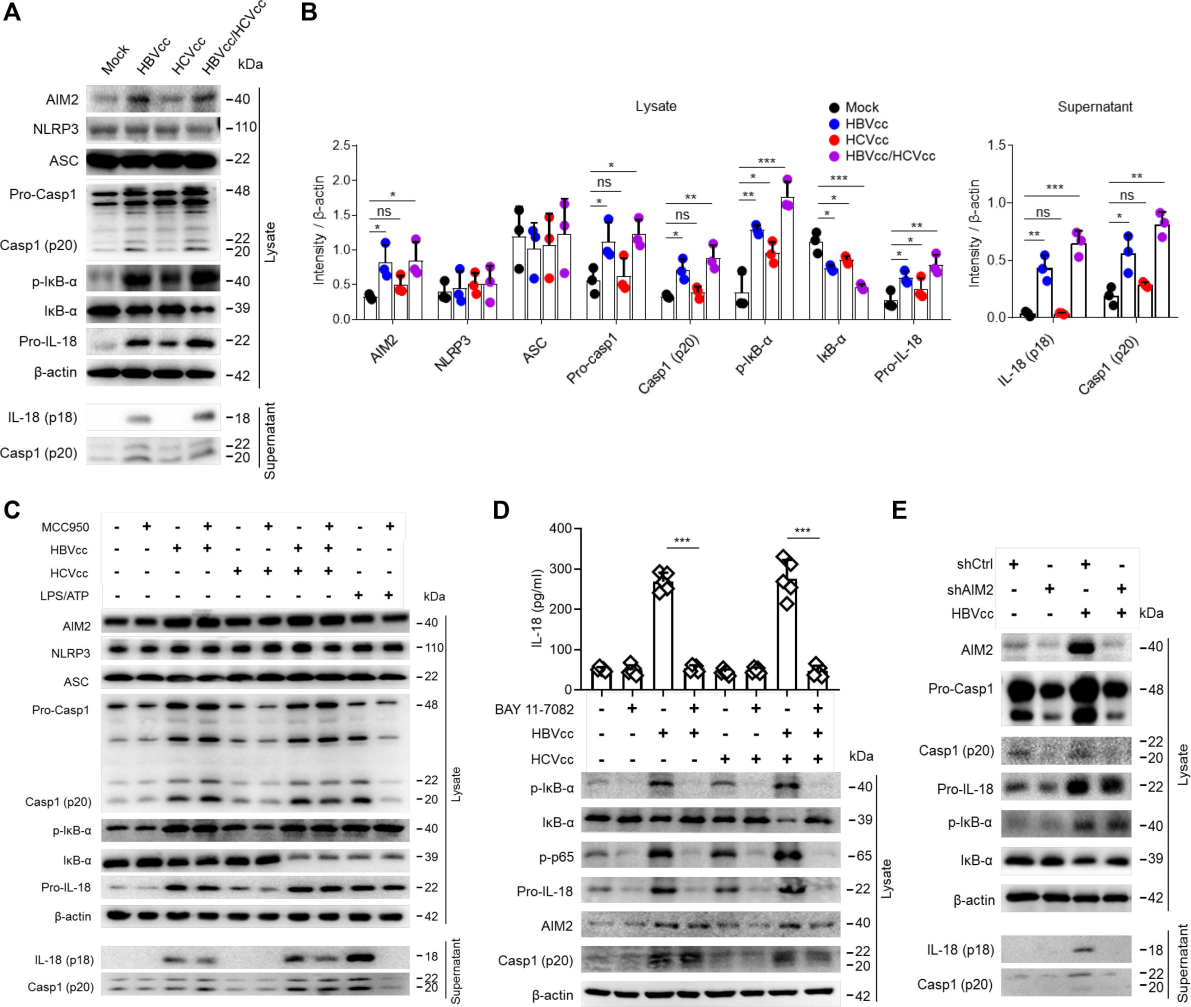
HBV induces monocytes IL-18 production by activating the AIM2 inflammasome. Purified monocytes (*n* = 3 per group) were stimulated with HBVcc and/or HCVcc for 24 h; the expression of AIM2, NLRP3, ASC, caspase-1, IκB-α, phosphorylate-IκB-α, and pro-IL-18 in the cell lysate, and IL-18 and caspase-1 in the supernatant were examined by Western blot (**A and B**). Pretreated monocytes (*n* = 5 per group) with 10 µM MCC950 (NLRP3 inhibitor) were stimulated with HBVcc and/or HCVcc for 24 h or treated with LPS (400 ng/mL) for 4 h, followed by ATP (5 mM) for 1 h; the relative protein expression level was examined by Western blot (**C**). Pretreated monocytes (*n* = 5 per group) with 5 µM BAY 11–7082 (NF-κB inhibitor) were stimulated with HBVcc and/or HCVcc for 24 h, the expression of IL-18 was examined by ELISA, and the relative protein expression level was examined by Western blot (**D**). THP-1 cells of AIM2-knockdown or control were stimulated with HBVcc for 24 h, and the relative protein expression level was examined by Western blot (**E**). Mean with SD and *P*-value are displayed. Comparisons between groups were measured by *t*-test in D or one-way ANOVA in B. *P < 0.05, **P < 0.01, ***P < 0.001, *ns*, not significant.

Previous studies revealed that AIM2 expression was higher in acute HBV patients and that AIM2 expression positively correlated with the serum level of IL-1β and IL-18 (22). To test if HBV and HCV activate the AIM2 inflammasome in monocytes, purified monocytes from healthy donors were exposed to HBVcc, HCVcc, or both HBVcc and HCVcc together. The protein expression of AIM2, pro-caspase-1, caspase-1, pro-IL-18, IκB-α, and IκB-α phosphorylation was detected in cell lysates and supernatants by Western blot. A representative experiment is shown in Fig. 4A, and the summary and statistical analysis are shown in Fig. 4B. Exposure to HCVcc induced pro-IL-18 expression and promoted IκB-α phosphorylation and IκB-α degradation in cell lysates, but not IL-18 secretion into culture in supernatants. Exposure to HBVcc and HBVcc/HCVcc not only induced the protein expression of AIM2, pro-caspase 1, and pro-IL-18 and promoted IκB-α phosphorylation and IκB-α degradation in cell lysates, but also promoted the secretion of IL-18 into culture supernatants. Next, to test whether the AIM2 pathway is sufficient by itself for the HBV response or NLRP3 pathway is responsible for IL-18 production as well, an NLRP3 inhibitor (MCC950) was added to monocytes cultured with HBVcc and/or HCVcc, and LPS/ATP was used as a solid positive control. We observed that MCC950 inhibited LPS/ATP-induced caspase-1 and IL-18 expression, but did not affect HBVcc- and HBVcc/HCVcc-induced AIM2 inflammasome activation and mature IL-18 production (Fig. 4C).

We further investigated if HBV induced IL-18 synthesis via NF-κB and AIM2 signals. At first, monocytes were treated with the inhibitor of NF-κB (BAY 11–7082) and cultured with HBVcc and/or HCVcc. The results showed that BAY 11–7082 inhibited HBVcc-induced and HBVcc/HCVcc-induced IL-18 secretion of monocytes as detected by ELISA, as well as HBVcc-induced and HCVcc-induced IκB-α phosphorylation, IκB-α degradation, p65 phosphorylation, and pro-IL-18 expression in cell lysates detected by Western blot, but not the expression of AIM2 and caspase-1 p20 (Fig. 4D). Next, AIM2 expression of THP-1 cells was knocked down using AIM2-targeting shRNA, and the THP-1 cells were exposed to HBVcc. We observed that AIM2 knockdown inhibited HBVcc-induced expression of AIM2, pro-caspase-1, and caspase-1, but did not affect IκB-α phosphorylation and pro-IL-18 expression in cell lysates (Fig. 4E).

Taken together, we concluded that both HBVcc and HCVcc activate NF-κB, which leads to the expression of pro-IL-18, but only HBV also activates the AIM2 inflammasome, leading to caspase-1 activity, cleavage and secretion of IL-18 cytokine.

### Monocytes phagocytose HBV and release dsDNA via lysosomes

**Fig 5:**
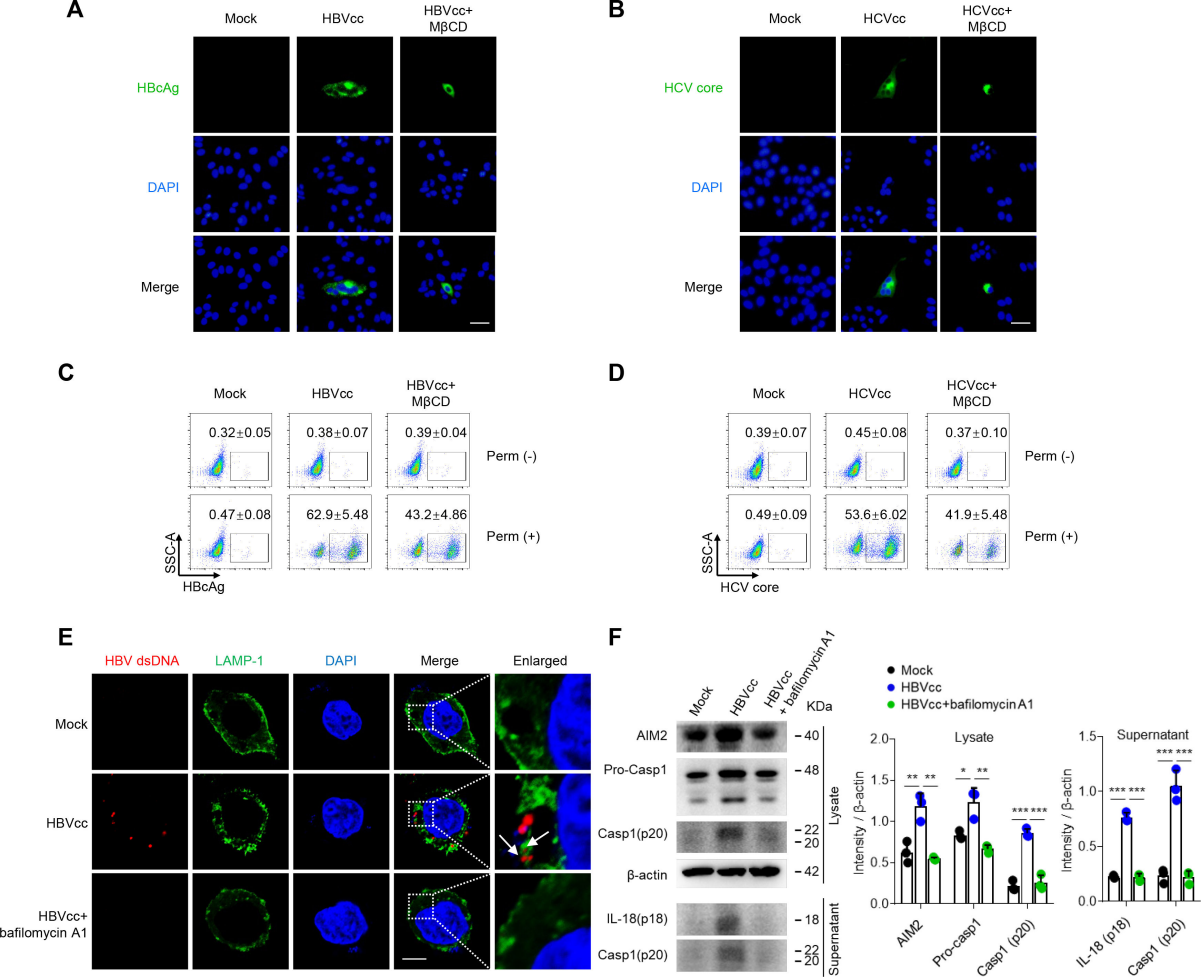
Monocytes phagocytose HBV and release dsDNA via lysosomes. Purified monocytes (*n* = 3 per group) were treated with or without phagocytosis inhibitor MβCD (5 mM) for 2 h, following with HBVcc or HCVcc for 12 h. HBcAg and HCV core proteins were detected by immunofluorescence (**A and B**), and cell surface (Perm-) and intracellular staining (Perm+) were detected by flow cytometry (**C and D**). Purified monocytes (*n* = 3 per group) were treated with or without lysosome inhibitor bafilomycin A1 (100 nM) for 12 h, following with HBVcc for 12 h. HBV dsDNA and LAMP-1 were stained using fluorescence confocal microscopy (**E**), and the relative protein expression level was examined by Western blot (**F**). A and B, Scale bar = 50 µm; E, Scale bar = 5 µm. Mean with SD and *P*-value are displayed. Comparisons between groups were measured by one-way ANOVA. *P < 0.05, **P < 0.01, ***P < 0.001.

As a cytosolic innate immune receptor, AIM2 recognizes double-stranded DNA (dsDNA) released during cellular perturbation and pathogenic assault and leads to the assembly of the AIM2 inflammasome (21). We first assessed whether HBV/HCV proteins were detected in the monocytes or examined whether HBV/HCV were attached to the surface of the monocytes. Pure monocytes were cultured with HBVcc and HCVcc in the presence or absence of MβCD (an inhibitor of phagocytosis) for 12 h. The HBcAg and HCV core proteins on monocytes were examine by immunofluorescence (Fig. 5A and B) and flow cytometry (Fig. 5C and D). The results showed that both HBcAg and HCV core proteins were detected in the monocytes but not on the cell surface and that MβCD significantly inhibited the entry of HBVcc and HCVcc into monocytes.

Lysosomes are acidic organelles containing many degradative enzymes, including proteases, nucleases, peptidases, phosphatases, lipases, glycosidases, and sulfatases (29). To investigate how HBV releases viral dsDNA into the cytosol of the monocyte to induce AIM2 activation, monocytes were cultured with HBVcc in the presence or absence of lysosome inhibitor bafilomycin A1. HBV dsDNA and lysosomal marker LAMP-1 (lysosome-associated membrane protein 1) were evaluated by fluorescence *in situ* hybridization (FISH), and the expression of caspase-1 and IL-18 were examined by Western blot. We observed the co-localization between cytosolic HBV dsDNA with LAMP-1 and that bafilomycin A1 significantly inhibits the production and release of HBV dsDNA in monocytes (Fig. 5E) and HBV-induced AIM2 inflammasomes activation and IL-18 production (Fig. 5F).

Together, these data suggest that HBVcc and HCVcc were phagocytosed by monocytes but not attached to the surface of the monocytes, and HBVcc releases dsDNA into the cytosol of monocytes via lysosome.

### Monocytes sense HBV dsDNA via AIM2

**Fig 6:**
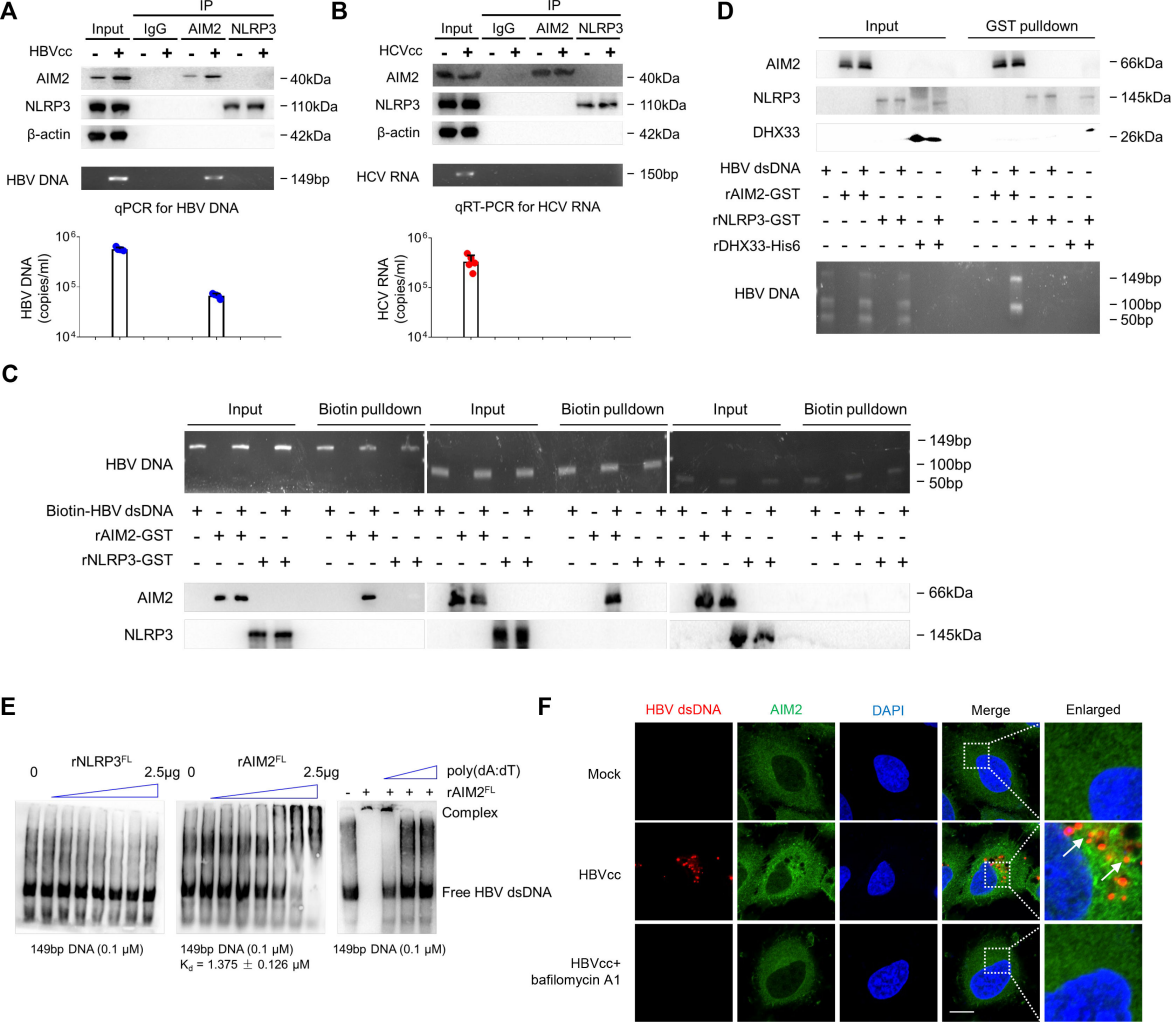
Monocytes sense HBV dsDNA via AIM2. Purified monocytes (*n* = 5 per group) were stimulated with HBVcc or HCVcc for 24 h, NLRP3 or AIM2 was precipitated from the cell lysates, and lysate and immunoprecipitation results were shown by Western blots (A and B, upper panel). HBV DNA and HCV RNA extracted from the complex were quantified by qPCR and qRT-PCR, respectively. Agarose gel image of PCR products (A and B, lower panel). Biotin-streptavidin pull-down assays of the binding of different length HBV dsDNA to recombinant AIM2^FL^ or NLRP3^FL^ (**C**). GST pull-down assays of the binding of recombinant AIM2^FL^ or NLRP3^FL^ to different length HBV dsDNA; 50 bp HBV dsDNA was used as a negative control for AIM2 binding, and DHX33 was used as a positive control for NLRP3 binding (**D**). EMSA of biotinylated 149 bp HBV dsDNA binding with recombinant AIM2^FL^ in the presence or absence poly(dA:dT) or NLRP3^FL^ (**E**). Purified monocytes were treated with or without bafilomycin A1 for 12 h, following with HBVcc for 12 h; HBV dsDNA and AIM2 were stained using fluorescence confocal microscopy. Scale bar = 5 µm. Representative images from one donor out of three are shown (**F**). Mean with SD is displayed.

To examine if AIM2 recognizes HBV dsDNA directly, we assessed AIM2 and NLRP3 binding of HBV dsDNA and HCV RNA by immunoprecipitation of AIM2 or NLRP3 from monocytes cultured with HBVcc or HCVcc followed by PCR analysis of HBV DNA or HCV RNA bound to AIM2 and NLRP3. This approach revealed that AIM2 but not NLRP3 bound to HBV DNA (Fig. 6A), and neither AIM2 nor NLRP3 bound to HCV RNA (Fig. 6B). To further confirm that AIM2 binds with HBV dsDNA, we performed experiments to measure direct binding of recombinant AIM2 and NLRP3 to different length HBV dsDNA using DNA pulldown (Fig. 6C), GST pulldown (Fig. 6D), and EMSA (Fig. 6E); 50 bp HBV dsDNA was used as negative control for AIM2 binding (30, 31), poly (dA:dT) was reported as an AIM2-bound positive control as well in our culture system (Fig. S3), and DHX33 (a member of DExD/H-box helicase family, sensed cytosolic RNA and formed a complex with NLRP3) was used as a positive control for NLRP3 binding (32). The results showed that HBV dsDNA was bound to AIM2, and the binding affinity of recombinant AIM2 with 149 bp HBV DNA is K_d_ = 1.375, but not NLRP3. We observed that poly(dA:dT) blocks the binding of HBV dsDNA to AIM2. To demonstrate how HBV dsDNA moves from lysosome to cytosol and stimulates AIM2-mediated caspase activation, the FISH experimental results showed the co-localization of HBV dsDNA and AIM2 in the cytosol of monocyte exposed to HBVcc, and bafilomycin A1 significantly inhibited the production of HBV dsDNA and AIM2 activation in monocytes (Fig. 6F). These data indicate that direct AIM2 recognition of HBV dsDNA leads to the assembly of a large multiprotein oligomeric inflammasome, and AIM2 inflammasome assembly in monocytes leads to the secretion of IL-18.

### HBV-induced AIM2 effect in the presence and the absence of HCV infection

**Fig 7:**
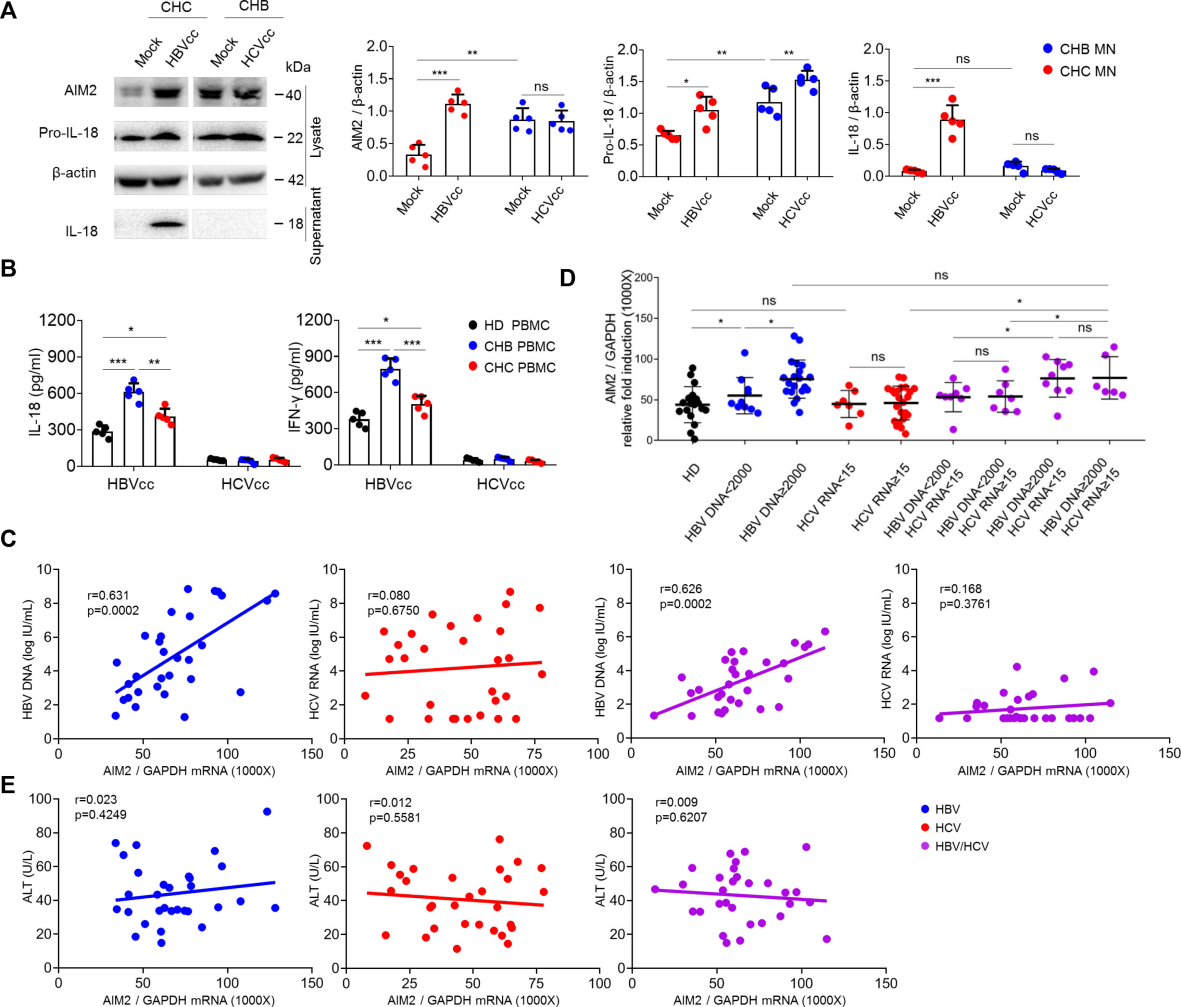
HBV-induced AIM2 effect in the presence and the absence of HCV infection. Purified monocytes (*n* = 5 per group) from CHC and CHB patients were stimulated with HBVcc or HCVcc for 24 h; the expression of AIM2 and pro-IL-18 in the cell lysate and IL-18 in the supernatant were examined by Western blots (**A**). Purified PBMCs (*n* = 5 per group) from HD, CHC, and CHB patients were stimulated with HBVcc and HCVcc for 24 h, and the expression of IL-18 and IFN-γ in the supernatant was examined by ELISA (**B**). Correlation between HBV DNA or HCV RNA levels and AIM2 mRNA in purified monocytes from HBV and HCV mono-infected and co-infected patients (**C**). Stratified analysis according to HBV and HCV viral load (**D**). Correlation between ALT and AIM2 mRNA in purified monocytes from HBV and HCV mono-infected and co-infected patients (**E**). Mean with SD and *P*-value are displayed. Comparisons between groups were measured by one-way ANOVA in A, B, and D. Spearman’s rank correlation coefficient was used to calculate correlations in C and E. *P < 0.05, **P < 0.01, ***P < 0.01, *ns*, not significant.

In the natural course of HBV/HCV co-infection, HCV superinfection may occur in the context of chronic HBV infection, or vice versa. To mimic HBV-induced and HCV-induced monocyte and NK cell activation during HBV and HCV superinfection in chronic HCV (CHC) and chronic HBV (CHB) infection, pure monocytes from CHB and CHC patients were challenged with the other virus. The protein expressions of AIM2, pro-IL-18, and IL-18 were detected in cell lysates and supernatants of monocytes by Western blot. The results showed that HBVcc increased the expression levels of AIM2, pro-IL-18, and IL-18 in monocytes from CHC patients. In contrast, HCVcc induced pro-IL-18 but did not manifest the other effects in monocytes from CHB patients (Fig. 7A). In addition, PBMCs from HD (Healthy Donor), CHB, and CHC patients were stimulated with HBVcc and HCVcc; we observed that HBVcc induced the production of IL-18 and IFN-γ in PBMCs from CHB, CHC patients, and HD, while HCVcc did not have these effects in PBMCs from CHB, CHC patients, and HD. Both IL-18 and IFN-γ production of PBMCs from CHB patients upon response to HBVcc are much more than that from HD and CHC patients (Fig. 7B).

To test whether the effect of HBV inducing AIM2 *in vitro* occurs really in HBV/HCV mono- and co-infection, we compared AIM2 expression of monocytes among HBV/HCV mono- and co-infected patients and HD. Monocytes were purified to determine the mRNA expression of AIM2 by qRT-PCR, and the relative fold induction was normalized to a house-keeping gene (GAPDH). The mRNA expression level of *AIM2* was positively correlated with HBV viral load in HBV mono-infection and HBV/HCV co-infection, but not with HCV viral load in HCV mono-infection and HBV/HCV co-infection (Fig. 7C). Based on viral load, above 2,000 IU/mL is defined as active HBV infection, and above 15 IU/mL is defined as active HCV infection as described previously (33). Further stratified analysis showed that the mRNA expression level of *AIM2* was significantly higher among the 20 HBV mono-infected and 15 HBV/HCV co-infected patients with HBV viral load >2,000 IU/mL than 10 HBV mono-infected and 15 HBV/HCV co-infected patients with HBV viral load <2,000 IU/mL and 18 HD, whereas HCV viral load (both ≥15 IU/mL and <15 IU/mL) among HCV mono-infection and HBV/HCV co-infection did not affect *AIM2* expression compared with HD (Fig. 7D). In addition, the mRNA expression level of *AIM2* was not correlated with ALT among HBV/HCV mono- and co-infected patients (Fig. 7E).

These results further argue that HBV initiates AIM2 inflammasome activation of monocytes leading to IL-18 synthesis, which contributes to NK cell IFN-γ production for the inhibition of viral replication in HBV mono-infection and HBV/HCV co-infection. Consistent with our conclusion, the level of AIM2 expression was tightly correlated with HBV levels, but not correlated with ALT.

## DISCUSSION

HBV and HCV are not directly cytopathic, and immune-mediated antiviral and inflammatory response determine both whether the virus is cleared and whether liver diseases are induced (34). In the case of spontaneous clearance of HCV or HBV infection, the immune response results in temporary liver injury, revealed by elevated levels of alanine aminotransferases (ALT)(34). Here, we found that HBV but not HCV induced an antiviral effect resulting from IFN-γ secretion by PBMCs. Blockade of IFN-γ signaling inhibited the HBV-induced antiviral effect, manifest as reduced STAT1 phosphorylation in hepatocytes. Numerous studies have shown PBMCs activation upon co-culture of HCV replicon cells requires close cell-to-cell contact, which involves sensing of HCV RNA by pDCs from secreted exosomes, but in trans-wells co-cultures a different mechanism is required (35 – 37). The biological relevance of these observations is that HBV induces antiviral effects in both HBV mono-infection and HBV/HCV co-infection. Following primary infection of adults with HBV, HBV-DNA rapidly decreases before the ALT level peaks by the 12th–16th week (38). A similar process was observed following the inoculation of a chimpanzee with HBV (39). This early virus control is probably sustained by non-cytopathic mechanisms (40). IFN-γ has been reported to be a key player in the mediation of non-cytopathic HBV eradication (41 – 43). Unlike HBV infection, studies of HCV infection in experimental chimpanzee models have demonstrated a high viral replicative capacity during the first days to weeks of infection, a phase that was followed by a plateau stage preceding the development of the host immune response (44, 45). Following primary exposure of the hosts to HCV, this virus maintains high replicative levels in the infected liver, resulting in the induction of IFNs and ISGs (46). The case of successful viral eradication in HCV infection and the suppression of HBV replication in HBV/HCV co-infection may be attributed largely to the IFN/ISG response in HCV-infected hepatocytes (12).

NK cells play an important role in early innate immune responses (47). In this study, we identified NK cells as the cell subset responsible for IFN-γ production in peripheral blood upon response to HBV but not HCV, and the antiviral significance of NK cells was demonstrated by a significant reduction of IFN-γ production and antiviral function when NK cells were depleted from PBMCs. Fisicaro et al. reported two intriguing cases of patients who successfully cleared HBV after the primary infection without any apparent elevation in ALT level and revealed that NK cells were activated and produced IFN-γ prior to the increase in HBV antigen-specific CD4^+^ or CD8^+^ T cells (48). These clinical data are consistent with our results from *in vitro* models, suggesting that NK cells play important roles in non-cytopathic viral clearance before adaptive immune responses are fully evoked.

Monocytes represent approximately 10% of leukocytes in human peripheral blood and also reside in or pass through the liver as precursors of macrophages, dendritic cells, and a subset of Kupffer cells. Here, we show that monocyte activation was responsible for the HBV-induced NK cells IFN-γ production, leading to an antiviral effect, and that the monocytes synthesize NK cell-activating signals, among which IL-18 is critical. These results reproduce several clinical studies, which have shown that the expression of IL-18 is significantly elevated during HBV infection (23, 49). Our previous studies found that both HBV and HCV activate TLR2/NF-κB signaling to induce the production of inflammatory cytokines in human monocytes and Kupffer cells (50, 51) and have addressed the impact of TLR signaling on the crosstalk between NK cells and Kupffer cells and revealed TLR-activated Kupffer cells synthesize and release IL-18 to promote NK cell IFN-γ production (52). However, the mechanism by which HBV induces IL-18 synthesis and release in monocytes remained unknown.

The AIM2 protein is an inflammasome receptor sensing cytosolic dsDNA expression in monocytes/macrophages and activates caspase-1 in an ASC-dependent manner, leading to release of the proinflammatory cytokine IL-18 (21), which induces NK cell activation resulting in an antiviral effect (20). We further found that both HBV and HCV activate NF-κB, which leads to the expression of pro-IL-18, but only HBV dsDNA activates the inflammasome via binding AIM2, leading to caspase-1 activity, cleavage, and secretion of IL-18 cytokine. To the best of our knowledge, this is the first description of HBV-induced activation of the AIM2 inflammasome signaling cascade in human monocytes leading to the secretion of IL-18. The AIM2 inflammasome is associated with HBV infection (22 – 24). Thus, AIM2 expression was higher in acute HBV patients, and AIM2 expression positively correlated with serum level of IL-1β and IL-18 (22). These clinical observations are consistent with our *in vitro* results.

Two distinct signals are required for the activation and release of IL-18 (53). The first signal is the activation of NF-κB in stimulated cells to induce transcriptional activation of *IL-18*. A second signal then activates AIM2 or NLRP3, ASC, and procaspase-1, resulting in inflammasome assembly to promote cleavage of procaspase-1 and secretion of IL-18. Cheng et al. reported that recombinant HBsAg inhibits LPS-mediated NF-κB signaling pathways and IL-18 production in THP-1 cell lines (54), and our previous study showed that HBsAg induces monocyte production of inflammatory cytokines via TLR2/MyD88/NF-κB signaling (50, 55). These indicate that both HBsAg and LPS activate NF-κB signaling and induce IL-18 expression, but HBsAg competitively inhibits LPS-mediated NF-κB signaling pathways and IL-18 production. It has been reported that HCV induces the activation of the macrophage NLRP3 inflammasome and release of IL-1β and IL-18 (56, 57). Exposed to HCV, human macrophages induce IL-1β and IL-18 secretion through NF-κB signaling rather than via the NLRP3 inflammasome (58). At present, these conflicting conclusions are difficult to reconcile. However, our results showed that HCV did not activate an inflammasome signaling cascade resulting in lack of monocyte IL-18 secretion although NF-κB was activated. A possible explanation is that HCV manipulates monocytes in a way distinct from its effects on macrophages.

**Fig 8:**
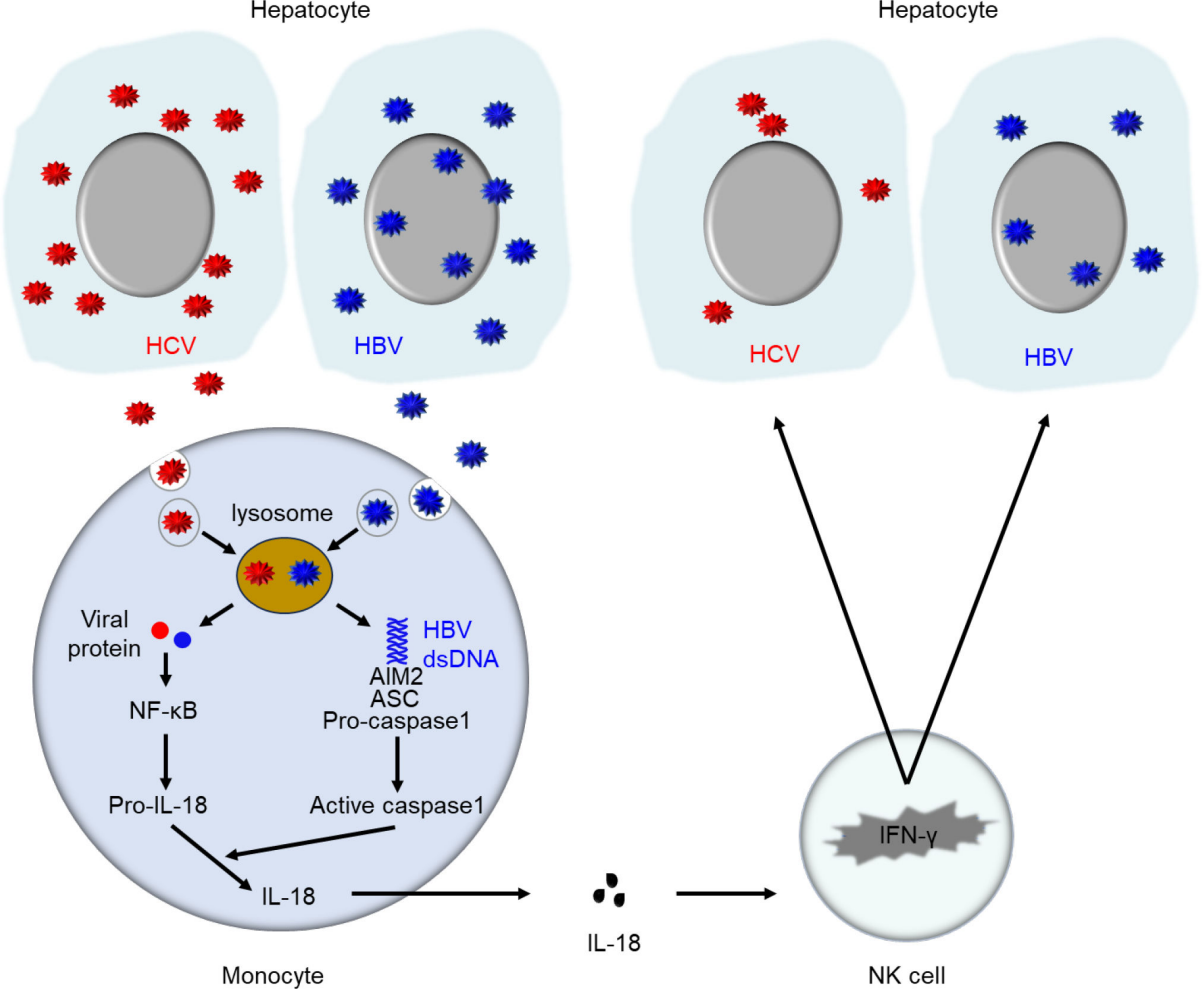
Schematic diagram of the innate immune response induced by HBV and HCV. Both HBV and HCV are phagocytosed by monocytes, and activate NF-κB, which leads to the expression of the pro-IL-18, but only nHBV dsDNA released via lysosome also activates the AIM2 inflammasome signaling cascade in monocytes, leading to caspase-1 activity, cleavage, and secretion of IL-18 cytokine. Since IL-18 induces IFN-γ in NK cells, this leads to an antiviral effect induced by HBV but not HCV.

In this study, our data lead us to propose a model of HBV-induced innate immune responses with cross-reactive effects on HCV (Fig. 8). Both HBV and HCV are phagocytosed by monocytes, and activate NF-κB, which leads to the expression of the pro-IL-18, but only HBV dsDNA released via lysosome also activates the AIM2 inflammasome signaling cascade in monocytes, leading to caspase-1 activity, cleavage, and secretion of IL-18 cytokine. Since IL-18 induces IFN-γ in NK cells, this leads to an antiviral effect induced by HBV but not HCV. Finally, we tested the predictions of this model against HBV/HCV mono-infected and co-infected patients, and the clinical observations and *ex vivo* experiments supported the model. This provides novel insights into the mechanism by which HBV induces antiviral effect in HBV mono-infection and HBV/HCV co-infection.

## MATERIALS AND METHODS

### Human subjects

Ninety treatment-naive HBV or HCV mono-infected and HBV/HCV co-infected patients were enrolled in this study (Table 1). We included patients (>20 years old) who were seropositive for HBsAg and/or the anti-HCV antibody (anti-HCV) for more than 6 months at the time of enrollment into the study. Venous blood was withdrawn for serum and peripheral blood mononuclear cells (PBMCs) collection. These studies were approved by the IRB of The First Hospital, Jilin University. Buffy coats from healthy donors were provided by the Changchun Blood Center, and informed consent was provided according to the protocols of the Changchun Blood Center.

**TABLE 1:** Clinical and virological characteristics of the patients

Characteristic	HD (*n* = 18)	HBV (*n* = 30)	HCV (*n* = 30)	HBV/HCV (*n* = 30)	*P* value
Age (years)	27.7 ± 6.1	30.7 ± 5.0	40.1 ± 8.2	39.2 ± 8.5	0.153
Sex (M/F)	10/8	17/13	16/14	16/14	0.356
Anti-HCV (–/+)	18/0	30/0	0/30	0/30	< 0.001
HBsAg (–/+)	18/0	0/30	30/0	0/30	< 0.001
HCV RNA (log IU/mL)	-	-	4.2 ± 2.4	1.8 ± 0.9	< 0.001
HBV DNA (log IU/mL)	-	4.9 ± 2.4	-	3.4 ± 1.5	0.031
ALT (U/L)	27.9 ± 19.2	42.4 ± 13.7	40.6 ± 17.8	42.9 ± 15.5	0.025
AST (U/L)	26.8 ± 10.3	35.2 ± 15.5	34.2.6±12.4	34.1 ± 10.3	0.038

### Cell culture-derived HBV

HepG2.2.15 cell lines were maintained at 37°C with 5% CO_2_ in Dulbecco’s Modified Eagle Medium (DMEM, Corning Life Science, Lowell, MA) containing 10% heat-inactivated fetal bovine serum (FBS), 100 U/mL penicillin and streptomycin. Cells were cultured and passaged in T75 flasks. Cell culture-derived HBV (HBVcc) production was obtained in HepG2.2.15 cell lines as previously described, with several modifications (50, 55). In brief, HepG2.2.15 cells were cultured in DMEM complete medium to induce HBV replication and virion production, and supernatant was collected every other day with culture medium replenishment for 15 days. The supernatant was centrifuged at 3,000 × *g* for 15 min to remove cell debris. The pooled supernatant was mixed with polyethylene glycol (PEG)−8000 powder (final concentration of 10%, Sigma-Aldrich) and gently rotated at 4°C for overnight. HBV particles were then precipitated by centrifugation at 1,500 × *g* for 30 min at 4°C and re-dissolved in sterile PBS or serum-free DMEM/F12 medium with 1% volume of the original supernatant samples. The concentrated virus stocks were aliquoted and stored at −80°C. For a control, HepG2 cell lines and supernatant were treated in the same way.

### Cell culture-derived HCV

Cell culture-derived HCV (HCVcc) production was obtained in JFH-1 Huh7.5 cell lines as described previously with modification (59). In brief, JFH-1 Huh7.5 cell lines were cultured in T75 flasks in DMEM complete medium for about 5 days. The supernatant was centrifuged at 3,000 × *g* for 15 min to remove cell debris. The pooled supernatant was concentrated by PEG8000, and the method was the same as for HBVcc. The concentrated virus stocks were aliquoted and stored at −80°C. As a control, Huh7.5 cell lines and supernatant were treated in the same way.

### Limulus amoebocyte assay for LPS contamination

Endotoxic contamination of HBVcc and HCVcc was assessed using QCL-1000 chromogenic end point assay (Combrex, Cottonwood, Arizona, USA) and found to be less than 1 pg/mL.

### Viral load measurements

HBV DNA and HCV RNA levels in serum, supernatant, or concentrated virus stocks were measured by standard curve using the EasyPure Viral DNA/RNA kit (TransGen Biotech, Beijing, China) according to the manufacturer’s instructions.

### Cell culture and co-culture

PBMCs, PBMCs depleted of monocytes or NK cells, and pure monocytes or NK cells were co-cultured in trans-wells with HepG2.2.15 or JFH-1 Huh7.5 cell lines as previously described (60). In brief, HepG2.2.15 or JFH-1 Huh7.5 cells were seeded in the lower chamber of a 12-well plate for 48 h and then co-cultured with PBMCs, PBMCs depleted of monocytes or NK cells, and pure monocytes or NK cells in the upper trans-well insert in the presence or absence of HCVcc (0, 10^3^, 10^5^, or 10^7^ copies/mL) or HBVcc (0, 10^3^, 10^5^, or 10^7^ copies/mL), HepG2.2.15 cells treated with entecavir (10 µM) or JFH-1 Huh7.5 cells treated with telaprevir (5 µM). After 24 h or 48 h, the supernatants were harvested for the detection of cytokines and viral load. Cell lines in the lower chamber were collected to detect HBV-DNA and HCV-RNA by qPCR and qRT-PCR and HBcAg and HCV core expression by Western blot. PBMCs were collected to assay IFN-γ expression by FACS analysis. Purified NK cells and monocytes were co-cultured for 24 h in complete medium in the presence or absence of HBVcc (10^5^ copies/mL) and/or HCVcc (10^5^ copies/mL), and Golgi plug (Sigma-Aldrich) was added for the final 4 h. NK cells were stained for intracellular expression of IFN-γ as previously described (55). Purified monocytes were cultured with HBVcc (10^5^ copies/mL) and/or HCVcc (10^5^ copies/mL) for 24 h as previously described (50). Cells were collected for Western blot, and the supernatants were harvested for cytokine detection by ELISA and detection of IL-18 and caspase-1 by Western blot.

### Enzyme-linked immunosorbent assay (ELISA)

The supernatants of cell cultures were collected in each experimental condition. The concentration of IFN-γ and IL-18 was measured by ELISA according to the manufacturer’s instructions.

### Flow cytometry

Staining of cells and analysis on a flow cytometer (FACS canto II; BD Biosciences) were done as described in our lab (59). V450 Mouse Anti-Human CD3 (Catalog: 560366, Clone: UCHT1), APC Mouse Anti-Human CD56 (Catalog: 555518, Clone: B159), PE-Cy7 Mouse Anti-Human CD16 (Catalog: 557744, Clone: 3G8), and PE Mouse Anti-Human IFN-γ (Catalog: 554701, Clone: B27) were obtained from BD Biosciences. In brief, PBMCs or NK cells were resuspended in staining buffer (0.5% BSA, 0.05% sodium azide in PBS) and pre-incubated with FcR blocking reagent (Miltenyi Biotec) for 15 min at 4°C. The cells were then simultaneously stained with suitable antibodies and then washed with staining buffer. For intracellular IFN-γ staining, cells were first fixed/permeabilized and subsequent IFN-γ staining performed according to the manufacturer’s protocol (Cytofix Buffer and Permeabilization Buffer III; Catalog:555028, BD Biosciences). The data acquired were analyzed with FlowJo Version 10 (Treestar software, Ashland, OR, USA).

### Quantitative reverse transcription PCR or quantitative PCR (qRT-PCR or q-PCR)

Total cellular RNA was isolated by EasyPure RNA Kit (TransGen Biotech). Total RNA was transcribed to cDNA by a reverse transcription kit (TransGen Biotech). Genomic DNA was extracted and purified from HepG2.2.15 cells by EasyPure Genomic DNA Kit (TransGen Biotech). Real-time PCR was performed with Power SYBR Green PCR Master Mix (TransGen Biotech) in an ABI StepOne Plus Real-Time PCR system (Applied Biosystems). The primer sequence pairs were designed using NCBI online primer blast software. Reactions were performed using 3 µL of cDNA in a 20 µL reaction volume and the following thermal cycle profile: 5 min for pre-denaturation at 94°C, 5 s for denaturation at 94°C, and 30 s for extension at 60°C, for 40 cycles.

### Immunofluorescence staining

To examine HBcAg or HCV core expression in HepG2.2.15 or JFH-1 Huh7.5 cells after co-culture with PBMCs, immunofluorescence staining was performed as previously described (61). Briefly, after 48 h of co-cultivation, an average sample of 2 × 10^5^ cells were seeded into a 35 mm confocal dish. After overnight, the cells were rinsed with PBS, fixed with 4% paraformaldehyde (PFA) at 4°C for 30 min, permeabilized with 0.5% Tween-20 for 10 min, blocked with normal 5%BSA (amresco) for 1 h, and sequentially incubated with HBcAg (1:200, Catalog: ab8637, Abcam) and HCV core (1:200, Catalog: MA1-080, Thermo Fisher Scientific) antibodies overnight at 4°C and secondary antibodies at room temperature for 1 h. Nuclei were counterstained with DAPI (1 µg/mL, Catalog: D1306, Thermo Fisher Scientific) for 5 min. Images were captured using a fluorescence microscope (Olympus IX51, Tokyo, Japan).

### Western blot analysis

Cells were lysed with RIPA buffer (Cell Signal Technology) supplemented with a proteinase/phosphatase inhibitor cocktail (Cell Signal Technology). After incubation on ice for 30 min, the lysates were centrifuged at 15,000 × *g* for 10 min at 4°C, and the protein-containing supernatant was collected for immunoblotting. After determining the concentration using the Bradford method, proteins were electrophoresed on SDS-PAGE gels and transferred to PVDF membranes (Merck Millipore, Darmstadt, Germany). The PVDF membranes were then blocked with 5% BSA, sequentially incubated with primary antibodies at 4°C overnight and secondary antibodies for 1 h at room temperature, and detected using ECL Chemiluminescent Substrate (Perkin Elmer). β-actin served as the internal control proteins.

Detection of protein levels in the supernatant was performed as previously described (62). In brief, cell culture supernatants were precipitated by adding an equal volume of methanol and 0.25 volumes of chloroform, vortexed and centrifuged at 20,000 × *g* for 10 min. The upper phase was discarded, and 500 µL of methanol was added to the interphase. This mixture was centrifuged at 20,000 × *g* for 10 min, and the protein pellets were dried at 55°C, resuspended in loading buffer, and boiled at 99°C for 5 min. As indicated, blots were incubated with rabbit monoclonal anti-human caspase-1 p20 (1:1000, Catalog: 3866, Cell Signaling Technology) and rabbit monoclonal anti-human IL-18 (1:1000, Catalog: 54943, Cell Signaling Technology).

### Plasmid construction and small hairpin RNA (shRNA)-encoding lentiviruses

Plasmid construction and lentivirus particles production were performed as previously described (61). The shRNA targeting AIM2 sequence was as follows: #1, GTGGTTTCTTAGAGGTAAA; #2, GCACCATAAAGGTTATTAA, and negative control, TAAGGTTAAGTCGCCCTCG. The sequences were assembled with the loop sequence (CTTCCTGTCAGA) and inserted into the pGreenPuro vector (System Biosciences, Mountain View, CA) according to the manufacturer’s instructions. Lentiviral vectors were transfected into HEK 293T cells using Lipofectamine 2000 (Invitrogen, Carlsbad, CA). Forty-eight hours after transfection, the lentivirus particles were harvested, concentrated, and stored at −80°C until use. For AIM2 knockdown in THP-1 cells, the infected cells were screened using 2 µg/mL puromycin (Sigma-Aldrich).

### Immunoprecipitation assay of AIM2/NLRP3 and virion DNA/RNA

Viral DNA/RNA-protein binding assay was performed using the protein precipitation assay as described previously with minor modifications (63, 64). Briefly, 1 × 10^7^ monocytes were stimulated with HBVcc or HCVcc. After 24 h, the media was replaced with PBS containing 1% PFA, and the cells were incubated for 10 min at room temperature. The crosslinking reaction was then quenched with 0.1M Tris pH 7.4 in PBS. The cells were lysed in 0.5% Triton X-100 lysis buffer. AIM2 or NLRP3 immunoprecipitation was performed by adding Protein G Agarose beads (Catalog: 37478, Cell Signaling Technology) and corresponding antibody to the lysates. After overnight incubation at 4°C, beads were washed twice with low salt, high salt, and lysis buffers. Complexes were eluted with a freshly prepared elution buffer (1% SDS and 0.1M NaHCO3, pH 8.0). Beads mixed with elution buffer was shaken on a vortex mixed for 15 min and then centrifuged at 10,000 x *g* for 3 min. Eluted samples were then de-crosslinked by adding 0.3M NaCl and incubating at 65°C for 4 h. This was followed by protein digestion using proteinase K at 45°C for 2 h. Viral DNA/RNA was finally purified using either acidic or basic phenol-chlorophorm-isoamyl alcohol. HBV DNA or HCV RNA bound to protein was quantified by qPCR or qRT-PCR with primers targeting the viral genome. The PCR products were also run on 1.5% agarose gels and imaged using a gel documentation system.

### GST/biotin pulldown assay

GST or biotin pulldown assay was performed as described previously with minor modification (65). Synthetic 50 bp (1,081–1,130 bp), 100 bp (1,685–1,784bp), and 149 bp (1,826–1,974 bp) HBV single-strand DNA oligos were biotinylated with Pierce DNA 3’ End Biotinylation Kit (Catalog: 89818, Thermo Fisher Scientific) according to the product instructions. HBV dsDNA was formed by annealing with the complementary strand. Aliquots of AIM2 recombinant protein (1 µg, GST tag, Sinobiological) or NLRP3 recombinant protein (1 µg, GST tag, Abnovus) were mixed with 2 pmol of biotinylated HBV dsDNA. The mixtures were incubated and precipitated GST-fused protein or biotin-labeled HBV dsDNA overnight at 4°C by GST tag-antibody (Catalog: 2625S, Cell Signaling Technology) and Protein G Agarose beads or Streptavidin magnetic beads (Catalog: IF9042, Engibody). The protein/DNA complexes were separated by centrifugation or using a magnet, protein samples were denatured by boiling in SDS sample buffer and then subjected to SDS-PAGE, and DNA samples were purified by phenol-chloroform-isoamyl alcohol and analyzed using qPCR to determine the enrichment of HBV dsDNA. The GST-fused protein and biotin-labeled HBV dsDNA amount in input samples were used as internal control.

### EMSA (Electrophoretic Mobility Shift Assay)

EMSA was performed as described previously (66). Briefly, AIM2 or NLRP3 recombinant protein (the highest dose is 2.5 µg and serially diluted twofold to generate seven doses in total) was mixed with 0.1 µM of biotinylated 149 bp HBV dsDNA and incubated at 4°C for 30 min. In competitive experiments, 2.5 µg AIM2 recombinant protein preincubated with poly(dA:dT) in different concentrations (0.05, 0.1, 0.2 µM) for 30 min and then incubated with 149 bp biotinylated HBV dsDNA. Then, the samples were resolved on 5% nondenaturing polyacrylamide gel prepared in 45 mM Tris-borate and 1 mM EDTA buffer. The specimens were electro-transferred onto a 0.45 µm Biodyne B nylon membrane (Catalog: 77016, Thermo Fisher Scientific) at 100V for 60 min at 4°C and cross-linked to the membrane by UV light for 10 min. Biotin-labeled HBV dsDNA was detected using the Chemiluminescent Nucleic Acid Detection Module (Catalog: 89880, Thermo Fisher Scientific). Unbound DNA was quantified as a proportion of total signal per lane with ImageJ software, and data were plotted against protein concentration to calculate the binding affinities indicated.

### FISH (Fluorescence *In Situ* Hybridization)

HBV DNA FISH was performed as previously described with modification (67). The FISH probe targeting the HBV strand was directly synthesized as oligonucleotides. The sequence of probe targeting HBV DNA is 5′-CTGCCTAATC ATCTCTTGTT CATGTCCTAC TGTTCAAGCC TCCAAGCTGT GCCTTGGGTG GCTTTGGGAC ATGGACATCG ACCCTTATAA AGAATTTGGA GCTACCGTGG AGTTACTCTC. The fluorescent probe was modified at the 5′ ends with an Alexa Fluor-594 dye. Treated cells were fixed with 4% PFA for 10 min at room temperature. Cells were then washed with PBS, permeabilized with 50% ethanol for 5 min, and dehydrated in 100% ethanol at −20 °C until use. Before hybridization, cells were rehydrated in 50% ethanol for 5 min, PBS for 10 min, and 4% PFA fixed the cells again. Cells were digested with RNaseA/H at 37 °C for 1 h. After washing with PBS and fixed (5 min) once more with 4% PFA fixed, specific probe was diluted in hybridization buffer and added to the slide, which was covered by coverslips and sealed with rubber cement. Hybridization was performed with preheating at 75 °C for 2 min and incubation at 40 °C for 3 h in a humidified chamber. Cells were blocked with 5% FBS and 0.1% Trixon-100 in PBS for 2 h at room temperature after label probe incubation and washing. Slides were then incubated with LAMP-1 antibody (Catalog: ab289548, Abcam) or AIM2 antibody (Catalog: ab93015, Abcam) followed by Alexa Fluor-488-labeled secondary antibody (Catalog: A-11029 or A-11034, Invitrogen). Cells were counterstained with DAPI for 5 min. After washing, images were acquired with a laser confocal microscope (Nikon, AXR Ti2-E, Japan) equipped with a 100× oil-immersion objective.

### Statistical analysis

The analysis of all data was performed with two-tail unpaired Student’s *t*-test or one-way ANOVA. Spearman’s rank correlation coefficient was used to calculate correlations. Data represent mean ± SD. *P*-values of <0.05 were considered statistically significant. Statistical analysis was performed using GraphPad Prism Version 8.01 (GraphPad Software, San Diego, CA).

## SUPPLEMENTAL MATERIAL

Fig.S1 to S4 jvi.01090-23-s0001.docx Supplemental text and figures.

ASM does not own the copyrights to Supplemental Material that may be linked to, or accessed through, an article. The authors have granted ASM a non-exclusive, world-wide license to publish the Supplemental Material files. Please contact the corresponding author directly for reuse.
