# Supplementary material for: Activation of AIM2 by hepatitis B virus results in antiviral immunity that suppresses hepatitis C virus during coinfection
Source: J Virol. 2023 Oct 3;97(10):e01090-23. doi: 10.1128/jvi.01090-23 (PMC10617567; doi:10.1128/jvi.01090-23)
Supplement: Fig.S1 to S4 — Supplemental text and figures. [file jvi.01090-23-s0001.docx]

Supplemental Materials for

**Title: Activation of AIM2 by Hepatitis B Virus results in anti-viral immunity that suppresses Hepatitis C Virus during coinfection**

**Authors:**

Yongqi Li,^a^ Yang Yang,^a^ Tianyang Li,^a^ Zhengmin Wang,^a^ Chunfeng Gao,^a^ Rilin Deng,^b^ Faxiang Ma,^a^ Xinyang Li,^a^ Licong Ma,^a^ Renyun Tian,^b^ Huiyi Li,^b^ Haizhen Zhu,^b^ Lei Zeng,^a^ Yanhang Gao,^c^ Guoyue Lv,^c^ Junqi Niu,^c^ Ian Nicholas Crispe,^d^ Zhengkun Tu^a,c*^.

**Affiliations:**

^a^ Institute of Translational Medicine, The First Hospital of Jilin University, Changchun, Jilin, China

^b^ Institute of Pathogen Biology and Immunology of College of Biology, Hunan Provincial Key Laboratory of Medical Virology, State Key Laboratory of Chemo/Biosensing and Chemometrics, Hunan University, Changsha, Hunan, China

^c^ Institute of Liver Diseases, The First Hospital of Jilin University, Changchun, Jilin, China

^d^ Department of Laboratory Medicine and Pathology, University of Washington, Seattle, WA, USA

***Correspondence to:** Zhengkun Tu, MD. Email: [tuzhengkun@jlu.edu.cn](mailto:tuzhengkun@hotmail.com);

**This PDF file includes:**

**Fig S1-4**

**Supplemental Methods**

**HBV infection of PBMCs.** HBV infection of HepG2-NTCP cells was conducted as previously described (1). Briefly, PBMCs or HepG2-NTCP cells as control were seeded on collagen-coated plates and pre-differentiated with 2.5% DMSO for two days prior to infecting with HBVcc in the presence of 4% PEG 8000 for 24h. The generated viruses were inoculated into HepG2-NTCP cells at 500 genome equivalents (GEq) per cell. The inoculum was removed by extensive washing with PBS and cells were cultured in the presence of 2.5% DMSO for indicated time. both intracellular and extracellular viral markers were analyzed.

**Flow cytometry for HBcAg and HCV core.** Intracellular HBcAg or HCV core in HepG2.2.15 cells or JFH-1 Huh7.5 cells were stained according to the manufacturer's protocol (Fix/Perm Buffer Set; Catalog:421403, Biolegend). The data acquired were analyzed with FlowJo Version 10 (Treestar software, Ashland, OR, USA).

**LPS/poly(dA:dT)-induced** **AIM2 inflammasome assay.** In order to activate the AIM2 inflammasome, purified monocytes were treated with 200ng/ml LPS for 3h, and transfected with 2μg/ml poly(dA:dT) using lipofectamine 2000 for 6h. The activation of AIM2 inflammasome was detected by western blot.

**Cell isolation and purification.** PBMCs were isolated based on the density gradient-based Ficoll-Paque. NK and monocytes were depleted from PBMCs by human CD56 and CD14 microbeads (Miltenyi Biotec, Bergisch Gladbach, Germany), respectively. Monocytes and NK cells were purified by magnetic cell sorting with CD14 microbeads and NK cells isolation kit (Miltenyi Biotec, Bergisch Gladbach, Germany), respectively. The cell depletion and cell purification were analyzed by flow cytometry. (Fig. S4).

**
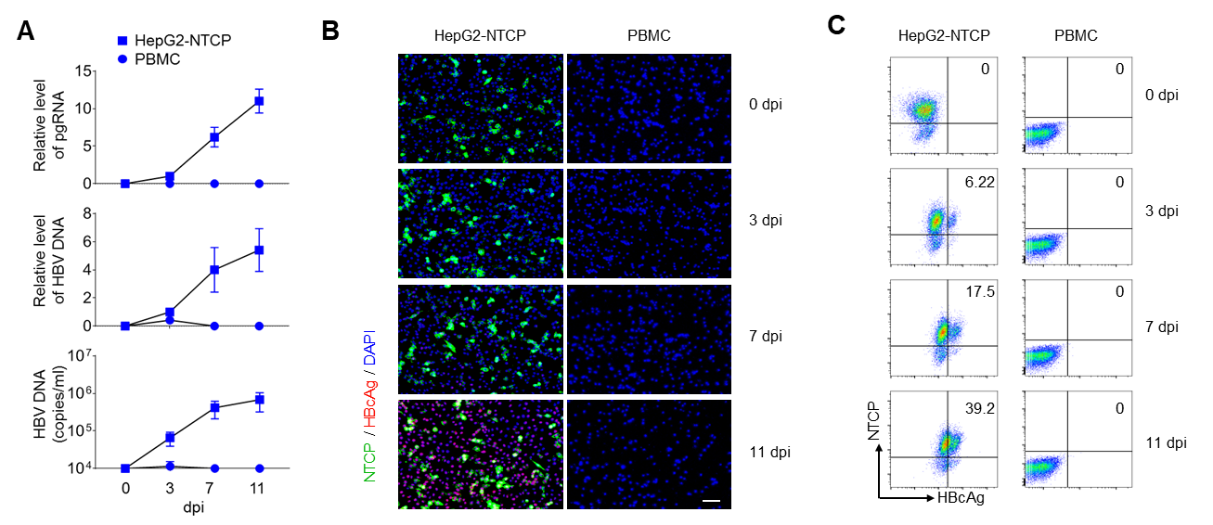
Fig S1** HBV infection of HepG2-NTCP, not PBMCs. HepG2-NTCP cells or PBMCs were infected with HBVcc at 500 GEq/cell. Intracellular HBV infection markers pgRNA and HBV DNA were determined by qRT-PCR or qPCR, respectively. Extracellular HBV DNA were determined by qPCR on day 0, 3, 7 and 11 postinfection (dpi) (A). HBcAg staining was performed by immunofluorescence (B) and flow cytometry (C). Scale bar = 100μm.


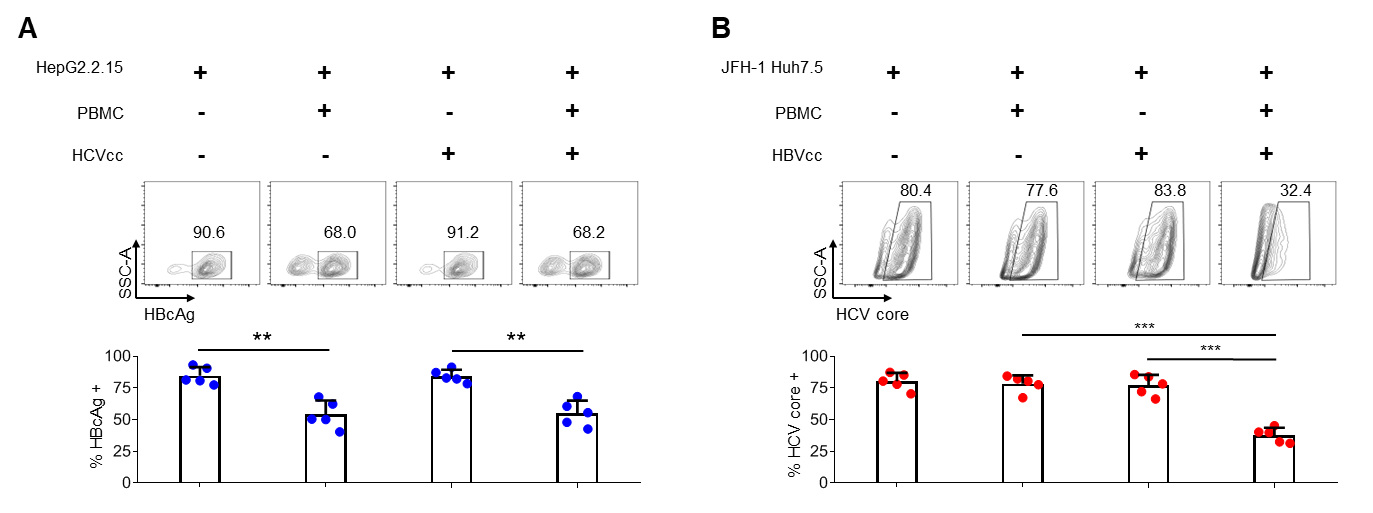
**Fig S2** HBV induces an antiviral effect in peripheral blood mononuclear cells, but HCV does not. HepG2.2.15 cells or JFH-1 Huh7.5 cells were co-cultured in trans-wells with or without PBMCs in the presence of HCVcc (10^5^ copies/ml) or HBVcc (10^5^ copies/ml) for 48h, HBcAg (A) and HCV core (B) expression were detected by intracellular staining. Mean with SD and *P* value are displayed. n=5 per group. Statistical significance was calculated using one-way ANOVA. ***P*<0.01, ****P*<0.001.


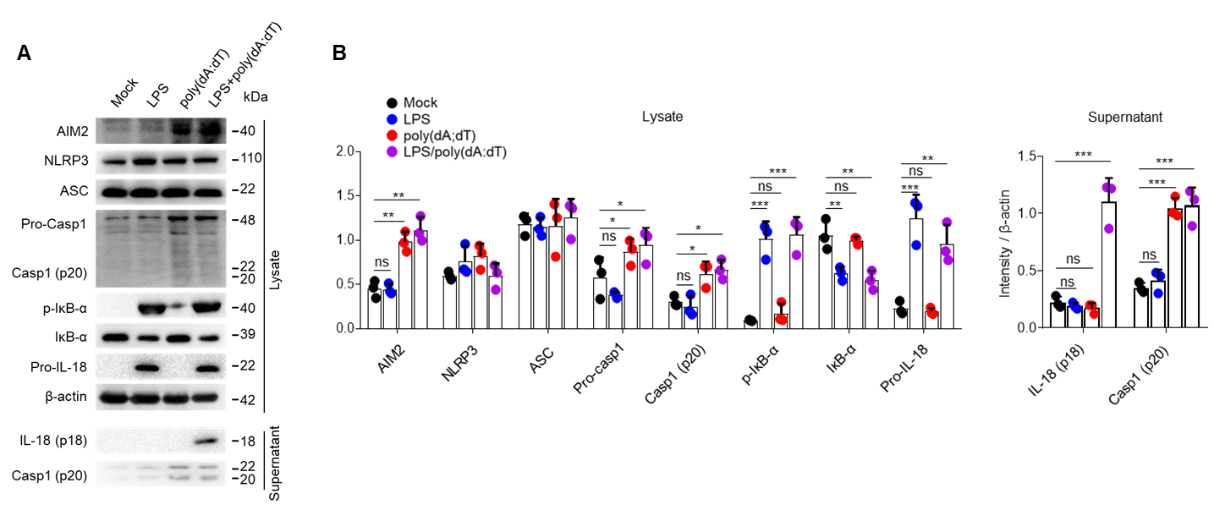
**Fig S3** LPS/poly(dA:dT)-induced the activation of AIM2 inflammasomes. Purified monocytes (n=3) were treated with LPS and/or transfected with poly(dA:dT), the expression of AIM2, NLRP3, ASC, Caspase 1, IκB-α, phosphorylate-IκB-α and pro-IL-18 in the cell lysate, and IL-18 and Caspase-1 in the supernatant were examined by Western blot.


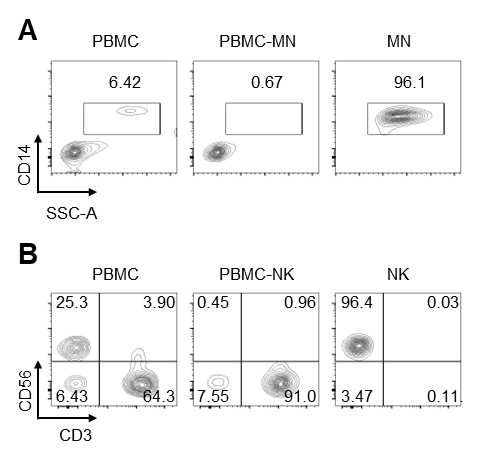


**Fig S4** The cell depletion and cell purification. The monocytes (A) and NK cells (B) depletion and purification were analyzed by flow cytometry.

**Supplemental references**

1. Ko C, Chakraborty A, Chou WM, Hasreiter J, Wettengel JM, Stadler D, Bester R, Asen T, Zhang K, Wisskirchen K, McKeating JA, Ryu WS, Protzer U**.** 2018. Hepatitis B virus genome recycling and de novo secondary infection events maintain stable cccDNA levels. J Hepatol 69**:**1231-1241. <https://doi.org/10.1016/j.jhep.2018.08.012>.
